# Newly graduated dentists’ knowledge of temporomandibular disorders compared to specialists in Saudi Arabia

**DOI:** 10.1186/s12903-020-01259-4

**Published:** 2020-10-07

**Authors:** Haila A. Al-Huraishi, Dalia E. Meisha, Wafa A. Algheriri, Wejdan F. Alasmari, Abdulmalik S. Alsuhaim, Amal A. Al-Khotani

**Affiliations:** 1 Department of Orofacial Pain and Jaw Function, Riyadh Specialized Dental Center, Riyadh, Saudi Arabia; 2 Scandinavian Center for Orofacial Neurosciences (SCON), Malmö/Huddinge, Sweden; 3 Department of Dental Public Health, Faculty of Dentistry, King Abdulaziz University, Jeddah, Saudi Arabia; 4 Department of Dentistry, John Hopkins Aramco Health Care Hospital, Dhahran, Saudi Arabia; 5 Features Dental Clinics, Riyadh, Saudi Arabia; 6 Deep Care Clinic, Riyadh, Saudi Arabia; 7 East Jeddah Hospital, Ministry of Health, Jeddah, Saudi Arabia

**Keywords:** Beliefs, General dentists, Knowledge, Orofacial pain, Specialists, Temporomandibular disorders

## Abstract

**Background:**

General dentists are often the first healthcare professionals to see patients with orofacial pain (OFP). OFP conditions associated with the temporomandibular joint are often confused with dentoalveolar disorders, which leads to mismanagement. The objective of this study was to evaluate the level of knowledge of temporomandibular disorders (TMD) among newly graduated dentists compared to OFP specialists in Saudi Arabia.

**Methods:**

This was a descriptive cross-sectional study utilizing an anonymous validated questionnaire assessing professional knowledge regarding TMDs in newly qualified dentists and OFP specialists. The questionnaire interrogated four domains including chronic pain/pain behavior, etiology, diagnosis/classification, and treatment/prognosis. OFP specialists were used as the reference group.

**Results:**

A total of 393 dentists participated, a response rate of 67.6% in newly graduated dentists and 77.3% in OFP specialists. The degree of agreement between newly graduated general dentists and OFP specialists was highest for the “chronic pain and pain behavior” domain. The consensus among specialists was highest for the “treatment and prognosis” domain and the least for the “chronic pain and pain behavior” domain.

**Conclusion:**

Newly graduated general dentists have limited knowledge of TMD in almost all domains compared to specialists. Given that a lack of knowledge of TMD can lead to clinical mismanagement, dental school curricula must address this important knowledge gap.

## Background

Despite the fact that temporomandibular disorders (TMD) are a common musculoskeletal disease affecting the temporomandibular joint and associated structures in the orofacial region [1], many dentists are unable to treat patients suffering from TMD. Until recently, general dentists often misdiagnosed the orofacial pain (OFP) of the temporomandibular joint (TMJ) with OFP associated with the dentoalveolar region, which results in incorrect management [2]. Many studies found that general dentists spent additional time and effort with patients suffering from TMD [3, 4]. However, it has been shown that patients suffering from OFP are misdiagnosed and poorly managed in primary healthcare centers, thus delaying treatment and referral [4]. In fact, over one-third of patients reporting positively to at least one of the three TMD screening questions were left untreated [5]. With an increasing number of dental visits due to pain in the orofacial regions, general dentists, who are considered primary caregivers, must be able to practice comprehensive dentistry independently, including identifying and diagnosing patients suffering from OFP problems [6]. In this regards, general dentists should be able to demonstrate professional responsibility along with interpersonal skills.

Moreover, studies have shown that dentists’ level of knowledge in the diagnosis and management of chronic, non-dental OFP was insufficient [2, 7, 8]. General dentists might be unaware of the continuous update of TMD taxonomies including the Diagnostic Criteria for Temporomandibular Disorders (DC/TMD) diagnostic system that was developed by the International Network for Orofacial Pain and Related Disorders Methodology (INfORM) [9]. Another explanation could be that some dental schools provide minimal theory on OFP or TMD and/or little clinical experience to such patients [10]. In Saudi Arabia, as in many dental schools, most dental colleges only teach TMD topics within courses such as prosthodontics, oral surgery, oral pathology, oral medicine, and oral diagnosis [7]. Furthermore, there is little or no clinical practice on actual TMD patients [11].

Together, newly graduated dentists may have limited knowledge about TMD and may lack the clinical experience that would enable them to appropriately diagnose and manage patients with orofacial pain of the regional muscles and TMJ or properly identify cases that need to be managed by a specialist. Despite this potential clinical risk, few published studies have assessed dentists’ knowledge of TMD [7, 12–14]. A recent study reported that general dentists’ knowledge of chronic OFP was low compared to dental specialists [8]. Another study showed that knowledge of TMD in children and adolescents is low in general dentists in Saudi Arabia compared to Swedish orofacial pain specialists [7]. Based on our review of the literature, there has been no assessment of the knowledge of newly graduated dentists about TMD in Saudi Arabia. Therefore, the objective of this study was to evaluate the level of knowledge regarding TMD in newly graduated dentists compared to OFP specialists in Saudi Arabia. We hypothesized that there is less consensus among newly graduated Saudi dentists in their knowledge regarding TMD compared to the reference group, Saudi OFP specialists.

## Methods

### Study design and setting

This was a descriptive cross-sectional study utilizing an anonymous validated questionnaire assessing dentists’ knowledge regarding TMDs [12]. The questionnaire was distributed to newly graduated dentists and OFP specialists in Saudi Arabia from April to June 2018. The Institutional Review Boards of King Abdul-Aziz Medical City (approval number H01-R-012) and King Fahad Medical City (approval number 18-132E) approved the study protocol. The cover page of the questionnaire noted that participants’ informed consent was implied by completing the questionnaire and that respondents who agreed to participate had the right to withdraw from the study at any time.

### Questionnaire

The questionnaire consisted of two sections with closed-ended questions and was administered in English. The first section asked about demographics including gender and number of years in practice after completing postgraduate training that led to OFP specialization for the specialists. The second section included 27 statements rated using a 5-point Likert scale (strongly agree, agree, neutral, disagree, and strongly disagree) to assess knowledge regarding TMDs. Twenty-two statements were adopted from validated questionnaires [12] used previously in the Saudi context [7]. Five new statements were formulated and added from the recent literature to be consistent with new updates in clinical practice [9]. In particular, recent studies have emphasized the importance of reporting comorbid conditions with TMJ dysfunction [15–17]. The five new statements were: “TMD pain is often associated with a clicking sound of the joint and/or restricted mouth opening”; “TMD pain is aggravated/relieved by jaw motion”; “Examination of neck muscles and TMJ in patients with chronic orofacial pain is important”; “Patients with rheumatoid arthritis should be asked for any TMD symptoms”; and “Migraine can cause or is comorbid with facial/jaw pain” [9, 15]. The content and clarity of the new statements were assessed by two specialists licensed in orofacial pain by the Saudi Commission for Health Specialties in Saudi Arabia, the licensing body for dentists. The questionnaire covered four main domains: chronic pain and pain behavior (3 statements), etiology (8 statements), diagnosis and classification (7 statements), and treatment and prognosis (9 statements) (Appendix A. Questionnaire).

### Participants

We invited all newly graduated dentists (556 dentists) who graduated in 2018 from all dental schools in Riyadh, Saudi Arabia to participate in the study. The participants were at the internship program which is the formal one-year paid comprehensive general dentistry experience. The questionnaire was distributed to the dentists in paper format during the monthly meeting of interns at each dental school in the Riyadh region. The same questionnaire was distributed to all orofacial pain specialists registered with the Saudi Commission For Health Specialties (SCFHS). This group was considered the reference group. The total registered number of individuals in the reference group at the start of the study was 22 specialists.

### Statistical analyses

Statistical analyses were performed using IBM SPSS Statistics (version 24.0, 2016, IBM Corp., Armonk, NY, USA). Categorical variables were expressed as percentages and numbers. Rating scores were expressed as medians and percentiles. Chi-square tests, Fisher exact tests, and Mann-Whitney tests were used to assess for differences between orofacial pain specialists and newly graduated general dentists. Score differences between specialists and newly graduated general dentists were analyzed using quantile regression. Quantile regression was performed using RStudio Team (version 1.1.218, 2016, RStudio: Integrated Development for R; RStudio, Inc., Boston, MA, USA). The statements were considered to show consensus if more than 75% of respondents either agreed (score of 4 or 5) or disagreed (score of 1 or 2). For statements that the OFP specialists agreed with, we analyzed the 25th percentile; if the OFP specialists disagreed with the statement, we analyzed the 75th percentile. The quantile regression model included percentiles of the scores and the gender. The OFP specialists group was used as the reference group against the newly graduated general dentists. *P* values of less than 0.05 were considered statistically significant.

## Results

### Demographics and response rate

**Fig. 1:**
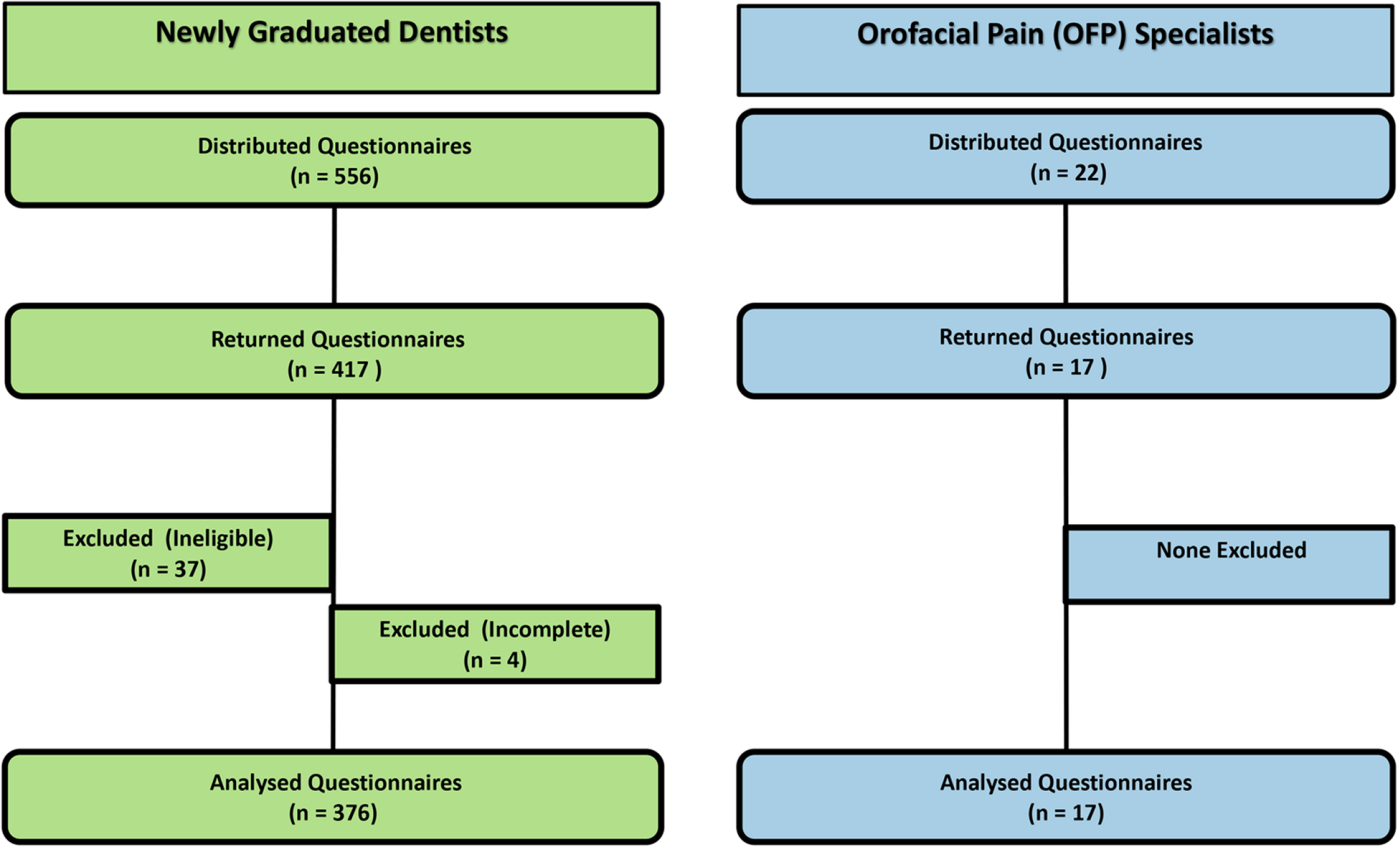
Flow diagram showing the number of questionnaires included in this study

A total of 393 dentists participated in this questionnaire, a response rate of 67.6% (376 participants) for newly graduated general dentists and 77.3% (17 participants) for OFP specialists (Fig. 1). Rating scores were not normally distributed as assessed by Shapiro-Wilk’s test (*p* < 0.0001). Table 1 shows the demographic characteristics of respondents. The proportion of male dentists was higher than female dentists (M:F ratio of 1.6:1). Eighty-eight percent of OFP specialists had over 3 years of experience.

**Table 1:** Demographic characteristics of responding orofacial pain (OFP) specialists and newly graduated general dentists

Variables	Total (*n* = 393)	OFP specialists (*n* = 17)	Newly graduated general dentists (*n* = 376)
	Percentage (n)	Percentage (n)	Percentage (n)
**Gender**			
Male	61.1% (240)	82.4% (14)	60.1% (226)
Female	38.9% (153)	17.6% (3)	39.9% (150)
**Years of practice**			
1–2 years		11.8% (2)	
3–4 years		41.2% (7)	
5–6 years		17.6% (3)	
6 years or more		29.4% (5)	

*P* value of Chi-square or Fisher exact test

### Reference group

The additional certification in OFP obtained by the specialists varied, as it included the American Board of Orofacial Pain (35.3%), advanced training or certification in OFP (35.3%), fellowship (11.8%), and Swedish Board (5.9%) certification. All specialists obtained their OFP certification from either the United States or Europe. The OFP specialists practicing in Saudi Arabia showed consensus for 74% of statements. The consensus among specialists was highest for the “treatment and prognosis” domain (77.8%), followed by the “etiology” domain (75%) and the “diagnosis and classification” domain (71.4%). The lowest consensus was for the “chronic pain and pain behavior” domain (66.7%).

### Degree of agreement

OFP specialists’ opinions differed significantly from newly graduated general dentists’ responses for 22 of the 27 statements (81.5%), as shown in Table 2. In the “chronic pain and pain behavior” domain, OFP specialists were consistently more likely to agree with the statements compared to the newly graduated general dentists. In this domain, there was consensus between specialists for two out of three statements. Specialists were more inclined to believe that sleep disturbances and depression are common symptoms in patients with OFPs. In the “etiology” domain, OFP specialists and newly graduated general dentists agreed on only one statement: “Oral parafunctional habits are often significant in the development of chronic TMD”. Specialists were less likely to believe that TMJ clicking is a serious symptom and that “headache is commonly related to psychological or social factors” compared to newly graduated general dentists.

**Table 2:** Comparison between orofacial pain (OFP) specialists (reference group) and newly graduated general dentists rating scores to different statements (a score of 5 indicates “Strongly agree” and 1 indicates “Strongly disagree”)

Domain	Statements	OFP Specialists					Newly Graduated General Dentists			*P*-value Mann- Whitney Test	Quantile regression Coefficient
		25th	Median	75th	Agree or Disagree	Consensus Yes/No	25th	Median	75th		
**Chronic pain and pain behavior**	• Chronic pain is a somatic and a behavioral and social problem.	3	4	4	A	No	3	3	4	0.1	0
	• Sleep disturbances are common in patients with chronic OFP.	4	4	4.5	A	Yes	3	4	4	0.002*	−1.0*
	• Depression can be an important etiologic factor in chronic OFP.	3.5	4	5	A	Yes	3	3	4	0.002*	−1.0*
**Etiology**	• TMJ clicking is a serious symptom which often creates a painful condition.	1.5	2	2	D	Yes	2	3	4	< 0.0001*	+ 2.0*
	• Oral parafunctional habits are often significant in the development of chronic TMD.	3.5	4	4.5	A	Yes	3	4	4	0.4	−1.0
	• Stress is a very important factor in the development of chronic TMD.	4	4	5	A	Yes	3	4	4	0.003*	−1.0*
	• Pain is the most common reason to seek treatment of TMD.	4	4	5	A	Yes	2	4	4	< 0.0001*	−1.0*
	• Patients with TMD who clench/brux do so either during the day or at night, but not both.	1.5	2	3	D	No	2	3	4	< 0.0001*	+ 1.0*
	• Headache is commonly related to psychological or social factors.	2	2	4	D	No	3	4	4	0.003*	+ 1.0*
	• Patients with rheumatoid arthritis should be asked for any TMJ symptoms.	4	4	5	A	Yes	3	4	4	< 0.0001*	−1.0*
	• Migraine can cause or is comorbid with facial/ jaw pain	4	4	5	A	Yes	3	3	4	< 0.0001*	1.0*
**Diagnosis and classification**	• TMJ disorders pain is often associated with a clicking sound of the joint and/or restricted mouth opening.	2	3	4	A	No	3	4	4	0.1	+ 1.0
	• Examination of neck muscles and TMJ with patients with orofacial chronic pain is important.	5	5	5	A	Yes	3	4	4	< 0.0001*	−2.0*
	• TMD pain is aggravated/relieved by jaw motion.	2.5	4	4.5	A	No	3	3	4	0.2	0.0
	• Reduced mouth opening capacity is almost never caused by TMJ arthritis.	1	2	2	D	Yes	2	3	4	< 0.0001*	+ 2.0*
	• Palpatory tenderness in the masticatory system and/or TMJ is the most important clinical sign of TMD.	4	4	5	A	Yes	3	3	4	< 0.0001*	−1.0*
	• TMD is more common amongst children with mixed dentition than amongst adult with permanent dentition.	1	2	2	D	Yes	2	3	4	< 0.0001*	+ 2.0*
	• Measuring mouth opening capacity is a reliable assessment method.	4	4	4.5	A	Yes	3	4	4	0.006*	−1.0*
**Treatment and prognosis**	• Occlusal grinding is a useful early treatment modality for TMD.	1	1	2	D	Yes	3	3	4	< 0.0001*	−2.0*
	• Orthodontic treatment can prevent the onset of TMD.	1	2	2.5	D	Yes	3	3	4	< 0.0001*	+ 2.0*
	• Orthodontic treatment can treat TMD.	2	2	3	A	No	3	3	4	< 0.0001*	+ 2.0*
	• Anti-inflammatory drugs are effective in the treatment of acute arthralgia.	4	4	5	A	Yes	3	4	4	< 0.0001*	−1.0*
	• The use of an occlusal splint is a good therapy in patients with TMD.	4	4	5	A	Yes	3	3	4	< 0.0001*	−1.0*
	• Relaxation-training is not an effective treatment for TMD.	1	1	2	D	Yes	2	3	4	< 0.0001*	+ 2.0*
	• Occlusal splints eliminate bruxism.	1	2	2.5	D	Yes	3	3	4	< 0.0001*	+ 2.0*
	• All individuals with TMJ clicking need treatment.	1	1	2	D	Yes	2	3	4	< 0.0001*	+ 2.0*
	• Counselling and behavioral therapy are the first line of treatment in patients which chronic TMD.	3	4	4.5	A	No	3	4	4	0.5	0.0

*statistical significance

**Fig. 2:**
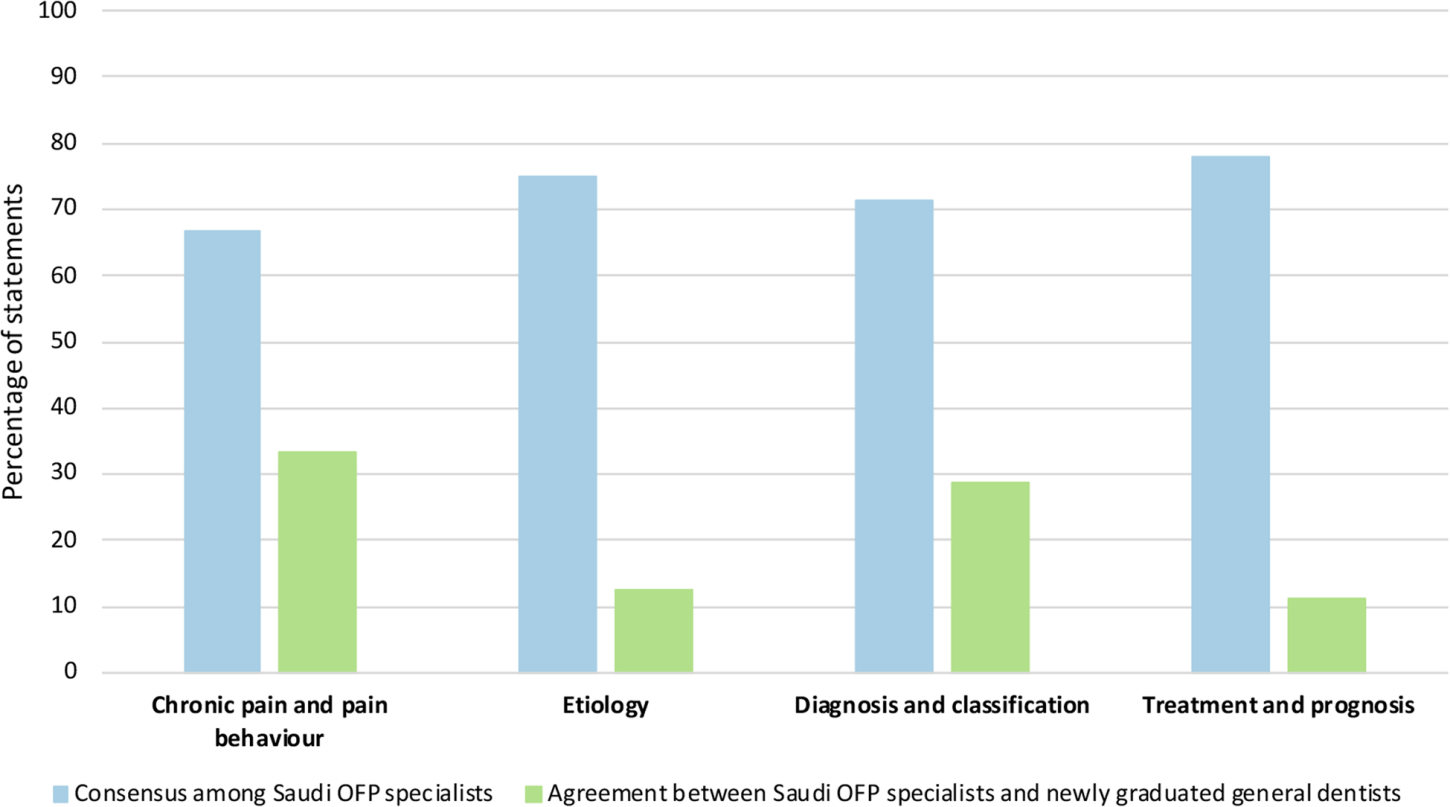
Proportion of statements showing consensus among Saudi orofacial pain (OFP) specialists and agreement between Saudi OFP specialists and newly graduated general dentists

Conversely, specialists were more likely to believe that stress and headache are important factors in the development of TMDs and that pain is typically what drives the patients to seek treatment and care. In the “diagnosis and classification” domain, specialists had a stronger belief in the need for comprehensive examination of the neck muscles/TMJ and measuring mouth opening compared to newly graduated general dentists. In the “treatment and prognosis” domain, specialists were less likely to believe in occlusal grinding and orthodontic treatment for the prevention and treatment of TMDs. In contrast, specialists had a stronger belief in the effectiveness of anti-inflammatory medication, occlusal splints, and relaxation training. The consensus among specialists was evidently higher than newly graduated general dentists, as shown in Fig. 2. Agreement between OFP specialists and newly graduated general dentists was highest for the “chronic pain and pain behavior” domain, although this was for only one-third of the statements.

## Discussion

Our findings support the hypothesis that knowledge of TMD in newly graduated general dentists showed less consensus in almost all domains compared to that of the reference group of OFP specialists. However, newly graduated general dentists differed the least with OFP specialists in the “chronic pain and pain behavior” domain, where there was agreement for no more than one-third of the statements. In agreement with our study, Al-Khotani et al. 2015 found that knowledge regarding TMD was inadequate not only among general dentists but also in oral and maxillofacial surgeons, orthodontists, and pedodontists in both Saudi Arabia and Sweden [7].

The consensus among Saudi OFP specialists (Reference group) was high for almost all domains. The consensus was highest in the “treatment and prognosis” domain. This finding is consistent with that of Al-Khotani et al. (2016), who reported that the consensus among Swedish OFP specialists was high in the “treatment and prognosis” domain [7]. However, in our study the consensus was the lowest in the “chronic pain and pain behavior” domain. This outcome is contrary to that of Al-Khotani et al. who found the lowest consensus was in the “diagnosis and classification” domain [7]. Consensus among Saudi and Swedish specialists in the “treatment and prognosis” domain is not surprising, since Saudi OFP specialists obtained their postgraduate training from the United States or Europe and therefore their treatment approaches would be expected to be very similar [18, 19]. It has been reported that Swedish OFP specialists start to acquire their knowledge of these conditions in their undergraduate and postgraduate studies [19]. Continuing postgraduate education has also been shown to have a positive impact on the knowledge of Swedish dentists [19, 20]. Moreover, these findings can be justified in part by the use of classification systems including the Research Diagnostic Criteria of Temporomandibular Disorders (RDC/TMD) and the expanded Diagnostic Criteria for Temporomandibular Disorders (DC/TMD) taxonomy, which are popular among Swedish OFP specialists [21].

In contrast to other studies [7, 12], we found the second-highest agreement between newly graduated general dentists and specialists was in the “diagnosis and classification” domain. Gnauck et al. reported that Swedish general dentists were able to diagnose TMD patients before referring them to OFP specialists. Furthermore, TMD knowledge between newly graduated general dentists and specialists in our study differed significantly in the “etiology” domain, except for the statement: “Oral parafunctional habits are often significant in the development of TMD”. This agrees with Tegelberg et al., who reported that over half of general dentists’ answers were significantly different from OFP specialists in the “etiology” domain. They also suggested that education and continuing education courses would increase general dentists’ knowledge and help them to better diagnose and manage TMD problems [12]. It is expected that increasing knowledge about TMD problems would reduce the risks of chronic development and misdiagnosis of these disorders.

Regarding the “treatment and prognosis” and “chronic pain and pain behavior” domains, newly graduated general dentists differed significantly from OFP specialists in over three-quarters of the questions. Likewise, an Australian study reported a lack of knowledge among final-year undergraduates in pain mechanisms [22]. Another study reported that TMD cases were improperly managed in German hospitals [10]. Taken together, this lack of essential knowledge might lead to uncertainty in management and affect the success of treatment.

A recent Swedish study reported that approximately half of patients who responded positively to TMD screening questions did not receive treatment for their TMD problems [5]. Although dental professionals in Sweden received an exclusive TMJ training program [7], they failed to appropriately manage patients suffering from TMD, perhaps due to some unknown factors affecting the quality of healthcare. This discrepancy warrants further investigation.

When comparing the consensus among the OFP Saudi specialists in the current study with the consensus among Swedish OFP specialists in previous study [7, 12], agreement between the Saudi and Swedish specialists was identical for all domains except for the “etiology” domain. In contrast, Tegelberg et al. found a low degree of consensus in the “diagnosis and classification” domain compared to the other domains in OFP specialists [12]. With reference to the DC/TMD diagnostic criteria [9], the emergence of the standardized classification systems (DC/TMD) might be expected to help unify knowledge in this regard. When comparing the consensus among OFP specialists, Swedish specialists had a higher consensus than Saudi specialists for both the “treatment and prognosis” and “etiology” domains. These differences might suggest that there is a need to improve healthcare workers’ knowledge in TMD in Saudi Arabia by improving not only medical professional training but also by embracing specialized institutes with interdisciplinary management approaches [2]. In Sweden, OFP has been recognized as a specialty since 1993 by the Swedish National Board of Health and Welfare, which fosters the current suggestion. The most recent acknowledgment was “Orofacial pain” as a recognized specialty by the American Dental Association National Commission on Recognition of Dental Specialties and Certifying Boards in the United States in February 2020 [23]. It is worth mentioning that in 2020, the SCFHS officially recognized the OFP as a distinct specialty in dentistry in Saudi Arabia. Furthermore, Saudi OFP specialists who obtained their postgraduate training from abroad started to establish OFP clinics in big cities such as Riyadh, Jeddah, Makkah, and Jazan. The Saudi ministry of health (MOH) continue to support scholarships for dentists who wish to pursue their postgraduate education in OFP field in order to meet the country’s needs. To our knowledge until now, there is no separate curriculum for OFP in the undergraduate and postgraduate level in Saudi dental colleges.

The present study has several strengths. One strength is that all potential newly graduated dentists in both private and public dental colleges in the Riyadh region were invited to participate. The response rate was 67.6% and, moreover, the response rate of OFP specialists was 77.3%. Both response rates exceeded the threshold of 60% response rate recommended for medical research [24], so the risk of non-response bias can be considered to be low and supporting the validity of the study. Another strength was performing quantile regression, which allows for a better understanding of the relationships between variables outside the context of the mean, normal distribution, and linear relationship assumptions [25]. We also used a validated questionnaire, which allowed comparisons with Swedish specialists. Future research directions could be to compare the undergraduate curriculum and course offerings on TMD in Saudi dental schools and explore variability, if any, between them. Since we included all possible newly graduated dentists in Riyadh region, the findings of our study could be generalized to newly graduated dentists from dental schools with similar curricula. It is worth mentioning that most Saudi dental schools were established by elite international dental school leaders, their curricula were adopted from international standards, and recently few have sought European recognition or American dental accreditation. This implies that Saudi dental school curricula are similar to their international counterparts. However, we believe that there has been little periodic update of the undergraduate dental curriculum in Saudi Arabian dental schools on TMD, as supported by previous studies [7, 11]. One limitation to this study may have been adding five questions regarding TMD comorbid conditions to the validated questionnaire questions. However, the inclusion of important risk factors that co-occurred with TMD was crucial, by offering knowledge that would help in identifying patients with jaw dysfunction. Another limitation was not including newly graduating dentists from dental schools in other regions of Saudi Arabia. This might affect the generalizability of the study results.

## Conclusion

In conclusion, there was a low level of consensus regarding TMDs among newly graduated dentists compared to OFP specialists in Saudi Arabia, reflecting the current undergraduate curriculum and training in this area.

## Supplementary information

**Additional file 1.** Appendix A: Questionnaire.

## Data Availability

Dataset available from the corresponding author upon reasonable request from qualified investigators.

## Abbreviations

TMD: Temporomandibular disorders
OFP: Orofacial pain
TMJ: Temporomandibular joint
