# Supplementary material for: Newly graduated dentists’ knowledge of temporomandibular disorders compared to specialists in Saudi Arabia
Source: BMC Oral Health. 2020 Oct 7;20:272. doi: 10.1186/s12903-020-01259-4 (PMC7542860; doi:10.1186/s12903-020-01259-4)
Supplement: Supplementary file 1 — Additional file 1. Appendix A: Questionnaire. [file 12903_2020_1259_MOESM1_ESM.docx]

**Appendix A: Questionnaire**

**Section 1: Demographic Data**

| **Gender:** Male/ Female |  |
| --- | --- |
| **Year of Graduation:** | **University/College:** |
| **Are you an orofacial specialist (OFP)?** Yes/ No |  |
| **If yes, answer the following:** |  |
| - Number of years practicing the specialty: |  |
| - What type of OFP certification did you obtain? | |
| - Country of OFP certification program: | |

**Section 2:**

To which extent do you agree or disagree with the following statements:

|  | Domain | Statement | Strongly Agree | Agree | Neutral | Disagree | Strongly Disagree |
| --- | --- | --- | --- | --- | --- | --- | --- |
| 1 | Chronic Pain and pain behavior | Chronic pain is a somatic and a behavioral and social problem. |  |  |  |  |  |
| 2 |  | Sleep disturbances are common in patients with chronic orofacial pain (OFP). |  |  |  |  |  |
| 3 |  | Depression can be an important etiologic factor in chronic OFP. |  |  |  |  |  |
| 4 | Etiology | TMJ clicking is a serious symptom which often creates a painful condition. |  |  |  |  |  |
| 5 |  | Oral parafunctional habits are often significant in the development of chronic TMD. |  |  |  |  |  |
| 6 |  | Stress is a very important factor in the development of chronic TMD. |  |  |  |  |  |
| 7 |  | Pain is the most common reason to seek treatment of TMD. |  |  |  |  |  |
| 8 |  | Patients with TMD who clench/ brux do so either during the day or at night, but not both. |  |  |  |  |  |
| 9 |  | Headache is commonly related to psychological or social factors. |  |  |  |  |  |
| 10 |  | Patients with rheumatoid arthritis should be asked for any TMJ symptoms. |  |  |  |  |  |
| 11 |  | Migraine can cause or is comorbid with facial/ jaw pain. |  |  |  |  |  |
| 12 | Diagnosis and classification | TMJ disorders pain is often associated with clicking sound of the joint and/or restricted mouth opening. |  |  |  |  |  |
| 13 |  | Examination of neck muscles and TMJ with patients with chronic orofacial pain is important. |  |  |  |  |  |
| 14 |  | TMD pain is aggravated/relived by Jaw motion. |  |  |  |  |  |
| 15 |  | Reduced mouth opening capacity is almost never caused by TMJ arthritis. |  |  |  |  |  |
| 16 |  | Palpatory tenderness in the masticatory system and/or TMJ is the most important clinical sign of TMD. |  |  |  |  |  |
| 17 |  | TMD is more common amongst children with mixed dentition than amongst adults with permanent dentition. |  |  |  |  |  |
| 18 |  | Measuring mouth opening capacity is a reliable assessment method. |  |  |  |  |  |
| 19 | Treatment and prognosis | Occlusal grinding is a useful early treatment modality for TMD. |  |  |  |  |  |
| 20 |  | Orthodontic treatment can prevent the onset of TMD. |  |  |  |  |  |
| 21 |  | Orthodontic treatment can treat TMD. |  |  |  |  |  |
| 22 |  | Anti-inflammatory drugs are effective in the treatment of acute arthralgia. |  |  |  |  |  |
| 23 |  | The use of an occlusal splint is a good therapy in patients with TMD. |  |  |  |  |  |
| 24 |  | Relaxation –training is not an effective treatment of TMD. |  |  |  |  |  |
| 25 |  | Occlusal splints eliminate bruxism. |  |  |  |  |  |
| 26 |  | All individuals with TMJ clicking need treatment. |  |  |  |  |  |
| 27 |  | Counselling and behavioral therapy are the first line of treatment in patients which chronic TMD. |  |  |  |  |  |
